# Cone Density Is Correlated to Outer Segment Length and Retinal Thickness in the Human Foveola

**DOI:** 10.1167/iovs.64.15.11

**Published:** 2023-12-08

**Authors:** Niklas Domdei, Julius Ameln, Aleksandr Gutnikov, Jenny L. Witten, Frank G. Holz, Siegfried Wahl, Wolf M. Harmening

**Affiliations:** 1 Carl Zeiss Vision International GmbH, Aalen, Germany; 2 Department of Ophthalmology, University of Bonn, Bonn, Germany; 3 Institute for Ophthalmic Research, Eberhard Karls University Tübingen, Tübingen, Germany

**Keywords:** adaptive optics, cone photoreceptors, outer segment length, optical coherence tomography, foveal development

## Abstract

**Purpose:**

Assessment of the relationship between in vivo foveolar cone density, cone outer segment length (OSL), and foveal retinal thickness (RT).

**Methods:**

Foveolar cone density maps covering the central ±300 µm of the retina were derived from adaptive optics scanning laser ophthalmoscopy images. The corresponding maps of foveal cone OSL and RT were derived from high-resolution optical coherence tomography volume scans. Alignment of the two-dimensional maps containing OSL and RT with the cone density map was achieved by placing the location of maximum OSL on the cone density centroid (CDC).

**Results:**

Across 10 participants (27 ± 9 years; 6 female), cone density at the CDC was found to be between 147,038 and 215,681 cones/mm². The maximum OSL and minimum RT were found to lie between 31 and 40, and 193 and 226 µm, respectively. A significant correlation was observed between cone density at the CDC and maximum OSL (*P* = 0.001), as well as the minimal RT (*P* < 0.05). Across all participants, the best fit for the relationship between normalized cone density and normalized OSL within the central 300 µm was given by a quadratic function.

**Conclusions:**

Using optical coherence tomography–derived measurements of OSL enables to estimate CDC cone density and two-dimensional foveal cone density maps for example in patient eyes unsuitable for adaptive optics imaging. Furthermore, the observation of a fixed relationship between the normalized OSL and cone density points to a conserved mechanism shaping the foveal pit.

The cone photoreceptors of the foveola are the functionally most relevant photoreceptors for human vision. Embedded in the most distal layer of the retina, these light-sensitive cells sample the retinal image with a higher spatial density than anywhere else in the eye,^1^^,^^2^ and eye movements continuously redirect gaze such that objects of interest fall into the foveal center.^3^^,^^4^ Foveolar cones are supported by a specialized neuronal architecture of the surrounding tissue and downstream circuitry that conserves both spatial and chromatic information of the retinal image, enabling high-acuity daytime color vision.^5^

To monitor, preserve, and potentially restore foveolar photoreceptors in cases of retinal disease will be of prime importance, because the loss of these cells has dramatic consequences for vision in most everyday activities.^6^^,^^7^ Unfortunately, the specific cellular morphology of foveolar cones with thin, elongated outer segments (OSs) packed at maximum density has made these cells historically the most difficult to study, both in ex vivo^8^ and in vivo^9^ approaches. Although research-grade adaptive optics ophthalmoscopy can now directly image foveolar cones^10^ and follow their fate during disease progression^11^^,^^12^ and pharmacological intervention^13^ in the living eye, most clinical-grade imaging devices, such as optical coherence tomography (OCT), owing to limited lateral resolution, cannot. To promote widespread clinical applicability, it is thus desirable to identify direct or indirect markers of foveolar cone structure leveraging the superior axial resolution in OCT imaging.

Structurally, the cone photoreceptors of the foveola are mostly clear of any overlying neural tissue. Second- and third-order neurons migrated outward during development, forming the foveal pit, and retinal blood vessels are absent in a coincidental area known as the foveolar avascular zone.^14^ The peak packing density of the OSs is a consequence of cone migration toward a common center during maturation of the fovea.^15^ Interestingly, cone OS volume seems to be constant throughout the retina,^16^ which, at higher density, leads to their elongated morphology in the foveal center. Psychophysically, longer OSs have greater photopic light sensitivity, owing to a higher number of photo pigment- containing discs passed by the incoming light,^17^ and their smaller diameter serves fine visual acuity.^18^ Given the relationship between cone OS length (OSL) and diameter (and thus cell density) observed in histological preparations,^16^ it ought to be possible to estimate cone density from cross-sectional in vivo measurements of the retinal tissue at the foveal center, such as provided by OCT. Surprisingly, previous studies failed to demonstrate a firm relationship between in vivo measurements of OSL and spacing which may be attributed to either limited image resolution and noisy metric extraction,^19^^,^^20^ or limited sample size.^21^

In the present study, we compared fine measurements of foveolar cone density using adaptive optics scanning laser ophthalmoscopy (AOSLO) with measurements of cone OSL and retinal thickness (RT) derived from a prototype, clinical-grade high-resolution OCT imaging system and find good correlation across both modalities and metrics in healthy eyes.

## Methods

Eleven human participants (mean age, 27 ± 8 years; see Table), with no known ocular conditions, were recruited and underwent high-resolution imaging of the foveola of both eyes, using AOSLO and OCT. Before AOSLO imaging, cycloplegia was induced by instilling 1 drop of 1% tropicamide. A custom dental impression (bite bar) was used to immobilize and control the position of the head for AOSLO imaging. Written informed consent was obtained from each participant, and all experimental procedures adhered to the tenets of the Declaration of Helsinki, in accordance with the guidelines of the independent ethics committee of the medical faculty at the Rheinische Friedrich–Wilhelms–Universität of Bonn, Germany. Throughout the article, participants are referred to with a singular ID, reflecting an ascending order of maximum cone density in the dominant eye.

### AOSLO Imaging and Image Montaging

The central ±300 µm in both eyes of each participant were imaged using near-infrared light for imaging and wavefront sensing, filtered dichroically (788 ± 12 nm; FF01-788/12-25, Semrock, Rochester, NY) from the output of a supercontinuum laser light source (SuperK EXTREME, NKT Photonics, Birkerød, Denmark). Adaptive optics correction, run in a closed loop at approximately 25 Hz, consisted of a Shack–Hartmann wavefront sensor (SHSCam AR-S-150-GE; Optocraft GmbH, Erlangen, Germany) and a 97-actuator deformable mirror (DM97-08; ALPAO, Montbonnot-Saint-Martin, France) placed at a pupil conjugate. The imaging raster spanned a square field of 0.85° × 0.85° of visual angle. The light reflected from the retina was detected in a photomultiplier tube (H4711-50, Hamamatsu, Japan), located behind a confocal pinhole (0.5 Airy disk diameter). Photomultiplier tube signals were sampled by a field programmable gate array board (ML506; Xilinx, San Jose, CA), producing video frames with 512 × 512 pixels (spatial resolution, 0.1 arcmin of visual angle per pixel) at approximately 27 or 30 Hz. To ensure optimal image quality during recording, the pupil's position relative to the AOSLO beam was carefully maintained.^22^

To guide the participant's gaze to image selected retinal locations, a 6-arcmin square fixation target was produced by modulating the imaging beam intensity, flashing at 3 Hz. Videos were recorded at the preferred retinal locus of fixation and eight surrounding points, evenly spaced and centered on the perimeter of the central imaging field, covering a square area of approximately ±300 µm of the fovea centered on the preferred retinal locus of fixation. Optimal image quality was found by selecting the best video from 5 to 10 preferred retinal locus of fixation–centered videos recorded using different defocus settings of the deformable mirror. At surrounding locations, two or more videos were recorded as deemed necessary by image quality. All videos were 10 seconds long. Acquired AOSLO video frames were spatially stabilized by offline, stripwise image registration using a modified version of previously published software in Matlab (MathWorks Inc., Natick, MA).^23^ Frames and strips displaying incomplete stabilization (e.g., owing to poor image quality, eye blinks, or drying tear film) were automatically identified and deleted. The remaining strips of each video frame were averaged to obtain a single high-quality image. Such images were automatically aligned using a previously described registration software.^24^ Regionally aligned images were imported in Corel Photo-Paint (Cascade Parent Limited, Ottawa, Ontario, Canada), subjectively selected for best retinal structural quality, and manually blended to arrive at a single continuous foveal image montage.

### Cone Density Maps and Cone Density Centroid

**Figure 1.**
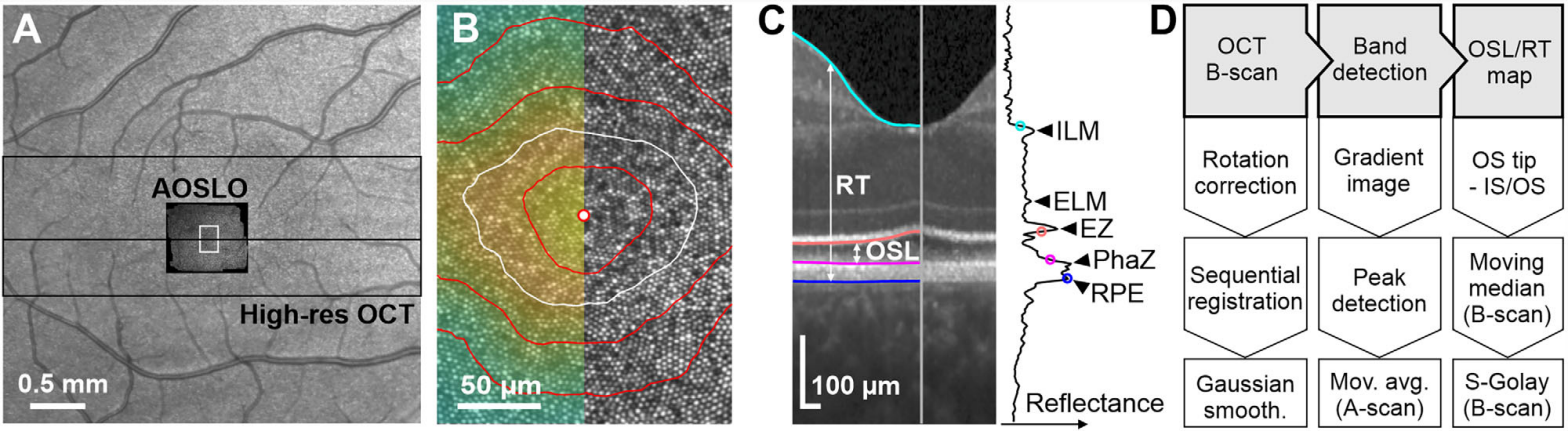
Image modalities, annotation, and analysis. (**A**) In each eye, AOSLO image montages covering the central ∼2° and 5° × 15° high-resolution OCT images were acquired and spatially aligned (shown here in participant P_09). (**B**) Crop from a central portion of the AOSLO montage (white box in **A**), centered on the CDC (centered marker). Contour lines represent relative cone density in 10% increments. The white contour line contains 20% of the highest cone densities. Colormap represents cone density. (**C**) Central OCT B-Scan (cropped) showing the segmentation derived through the processing pipeline. The OSL was defined as the distance between the offset edge of the ellipsoid zone (EZ) and the onset edge of the Phagosome zone (PhaZ) derived from single A-Scan reflectance profile.^27^ RT was defined by the distance between the internal limiting membrane (ILM) onset edge and the retinal pigment epithelium (RPE). (**D**) OCT image processing pipeline (for details see the Methods section).

The processing pipeline to generate continuous cone density maps and to determine the cone density centroid (CDC) has been described elswhere.^4^ In brief, the cone center locations in the final montage were labeled in a semimanual process by a single trained image grader: first, a convolutional neural network^25^ was used to annotate retinal images automatically and in a second step manually corrected using custom software in Matlab. Based on the labeled cone center locations, a Voronoi tessellation was computed (MATLAB functions: delaunayTriangulation, voronoiDiagram and voronoin). Each cone was regarded as occupying the space of each corresponding Voronoi cell. Angular cone density (cones/deg^2^) was computed at each image pixel by averaging the combined Voronoi area of the nearest 150 encircled cones around that pixel. Linear cone densities (cones/mm^2^) were computed by applying the individual retinal magnification factors of each eye,^26^ considering axial length, anterior chamber depth and corneal curvature, based on swept source biometry (IOLMaster 700, Carl Zeiss Meditech, Jena, Germany). Finally, the CDC was determined as the weighted centroid (MATLAB function: regionprops [‘WeightedCentroid’]) of the highest 20% of cone density values (Fig. 1B).

### OCT Imaging and Band Definitions

Spectral-domain OCT images of the fovea were recorded using a prototype high-resolution device (Heidelberg Engineering GmbH, Heidelberg, Germany). The axial resolution of this system was about 2 µm (in air). Near infrared imaging light (840 ± 68 nm) was provided by a super luminescent laser diode. The selected imaging field was 15° horizontal and 5° vertical containing 256 B-scans with 768 A-scans each (Fig. 1A). The resulting A- and B-scan lateral spacing was approximately 6 µm. From single A-scan reflectance profiles, cone OSL, OSL, was measured as the distance between the offset edge of the ellipsoid zone, defining the transition between the ISs and OSs (IS/OS), and the onset edge of the phagosome zone, defining the OS tips (Fig. 1C).^27^ RT was quantified by the distance between the internal limiting membrane onset edge and the retinal pigment epithelium. The outer nuclear layer including the ISs (ONL+) was given by the distance between the internal limiting membrane and the ellipsoid zone center in the reflectance profile.

### OSL/RT Map Generation and Multimodal Map Alignment

Two-dimensional OCT data maps (OSL and RT) were generated based on raw data files (.vol) processed by a custom pipeline prepared in Matlab software (Fig. 1D). First, OCT B-scan images were corrected for rotation and finely aligned to the preceding B-scan via sequential registration and smoothed with a gaussian filter. For band segmentation, each B-scan was converted into a gradient image, highlighting the edges of ellipsoid zone and phagosome zone for the subsequent peak detection. A moving average along the A-scan direction was used to smooth the B-scan's segmentation. Although the IS/OS and OS tip segmentation were processed by the described pipeline, the internal limiting membrane and retinal pigment epithelium segmentation were found by the OCT device's inherent software. In the final steps, the OSL or RT map was created by the difference between the according band segmentations across all B-scans. For further smoothing, a moving median and a Savitzky-Golay filter were applied in the B-scan direction. The final OSL and RT maps were scaled for linear space by each eye's retinal magnification factor. To estimate foveolar cone density maps from OCT data, the multimodal maps of OSL and RT were aligned with their corresponding maps of cone density by centering the maximum OSL at the CDC, assuming that both maps had a common center with minimal relative rotation.

### Statistics

All statistical analyses were performed in Matlab. Data correlations were calculated based on the F-test (function: regress) and confidence intervals computed using the functions: fitlm and predict. Data distributions were compared with a paired t-test after confirmation of normality by the Kolmogorov-Smirnov test. OCT maps of OSL and RT were up-sampled (function: imresize [‘bilinear’]) for a cone-wise comparison with density maps. When testing cone density estimations, cone density maps of the fellow eyes were down-sampled (function: imresize [‘bicubic’]) to match the resolution of the OCT data.

## Results

Using AOSLO imaging and advanced image analysis protocols, the foveolar cone mosaic in both eyes of 10 participants was resolved fully. The data of the 11th participant were excluded from the following analysis owing to incomplete resolution of all foveal cones at a small central patch in one of the eyes. This factor led to a likely overestimation of the true density in this eye, evident by comparison to estimates in the fellow eye, which had all cones resolved. Cone density maps of the dominant eyes were compared with OCT derived maps of OSL and RT (see Table and Supplementary Fig. S1).

**Table.** Individual Participant Data and Ocular Biometry

				Dominant Eye						Fellow Eye					
ID	Sex	Age	Dominant Eye	Axial Length (mm)	RMF (µm/Degree)	Density@CDC (Cones/mm²)	Max OSL (µm)	Min RT (µm)	Min ONL+ (µm)	Axial Length (mm)	RMF (µm/Degree)	Density@CDC (Cones/mm²)	Max OSL (µm)	Min RT (µm)	Min ONL+ (µm)
P_01 (BAK8015)	M	35	OD	25.00	304.1	147,038	31.1	212.2	147.4	24.87	301.8	142,715	30.4	202.6	140.8
P_02 (BAK1086)	F	21	OD	23.13	274.9	155,470	31.0	193.0	132.7	22.61	266.2	172,474	32.6	196.2	128.6
P_03 (BAK1028)	F	33	OD	22.85	270.8	163,552	33.9	215.3	144.0	22.68	268.8	162,431	34.1	212.6	144.5
P_04 (BAK1005)	F	30	OD	24.77	300.1	164,302	30.7	206.7	148.7	24.79	300.7	166,694	29.8	208.3	148.6
P_05 (BAK1091)	F	24	OS	23.97	287.6	179,852	36.5	221.3	152.9	24.42	295.4	181,739	33.4	223.0	159.5
P_06 (BAK8018)	F	29	OD	25.06	301.6	185,655	37.0	223.7	153.6	25.12	302.6	175,852	36.1	220.7	151.8
P_07 (BAK9019)	M	44	OD	23.68	285.0	197,180	34.0	226.0	161.9	23.57	285.6	200,453	34.9	223.8	160.2
P_08 (BAK1069)	M	15	OD	23.00	269.9	200,949	38.2	220.1	147.8	22.99	269.5	198,468	36.3	225.2	151.6
P_09 (BAK1070)	M	13	OS	22.88	269.5	210,271	39.6	217.1	153.5	22.81	268.6	206,225	38.9	209.2	144.6
P_10 (BAK1093)	F	25	OD	23.42	277.0	215,681	38.6	225.1	159.1	23.28	274.6	216,209	37.7	230.7	163.4
Mean ± STD		27 ± 9		23.78 ± 0.84	284.1 ± 13.0	181,995 ± 22,629	35.1 ± 3.2	216.1 ± 9.6	150.2 ± 7.8	23.71 ± 0.94	283.4 ± 14.7	182,326 ± 21,580	34.4 ± 2.8	215.2 ± 10.6	149.4 ± 9.9

RMF, retinal magnification factor.

**Figure 2.**
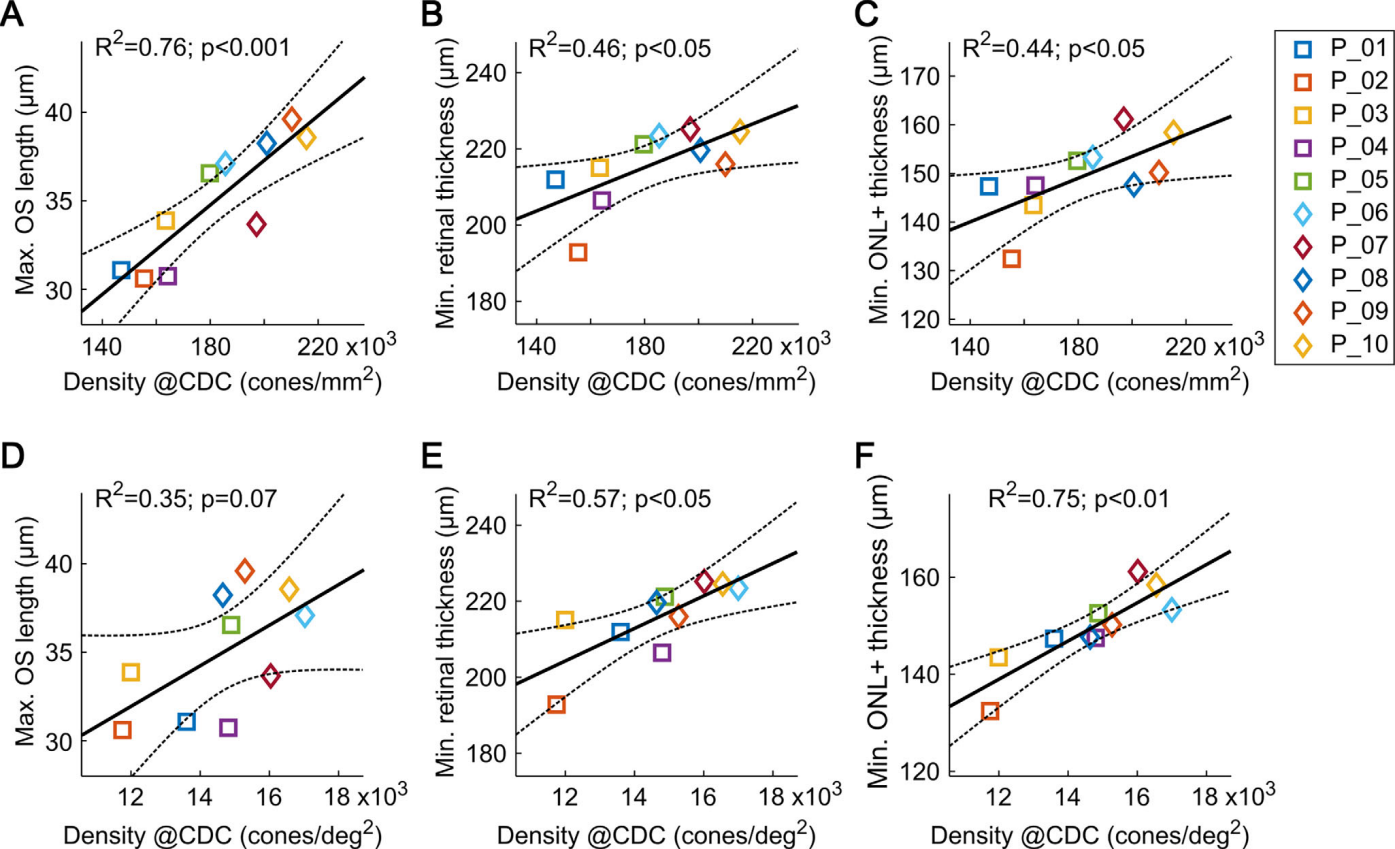
OCT-derived foveal metrics correlated to AOSLO-derived CD_CDC_, expressed in linear units of retinal space (**A**–**C**) and in units of visual angle (**D**–**F**). ONL+ is the distance from the internal limiting membrane to the phagosome zone. When analyzed across all participants (P_01-10, markers and colors), most correlations display statistical significance (F-test). R-square values of the linear fit to the data are given. Dashed lines indicate confidence intervals.

Across participants, cone density at the CDC (CD_CDC_) varied from 147,038 to 215,681 cones/mm² (181,995 ± 22,629 cones/mm²). The maximum OSL and minimum RT were found between 31 and 40 µm (35 ± 3 µm), and 193 and 226 µm (216 ± 10 µm), respectively. A significant correlation was observed between CD_CDC_ and maximum OSL (*P* = 0.001, F-test), as well as the minimum RT (*P* = 0.031) and ONL+ thickness (*P* = 0.036) (Figs. 2A–C). When cone density was converted into angular units (cones/deg^2^), cone density was significantly correlated with minimum RT (*P* = 0.011) and ONL+ thickness (*P* = 0.0012), but not with maximum OSL (*P* = 0.073) (Figs. 2D–F).

**Figure 3.**
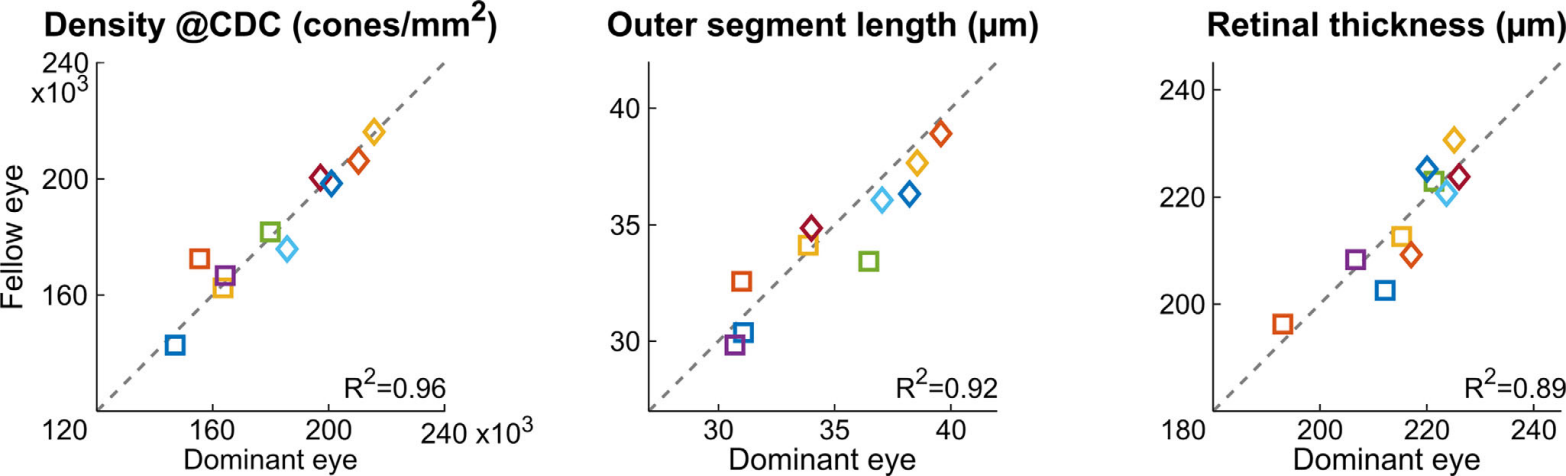
CD_CDC_, maximum OSL, and minimal RT in the foveal center were all highly correlated between fellow eyes. Dashed lines = unity, and participant markers are the same as in Figure 2.

The CD_CDC_, as well as maximum OSL, and minimum RT were highly correlated between dominant and fellow eyes, with an R^2^ of 0.96, 0.92, and 0.89. respectively (Fig. 3).

**Figure 4.**
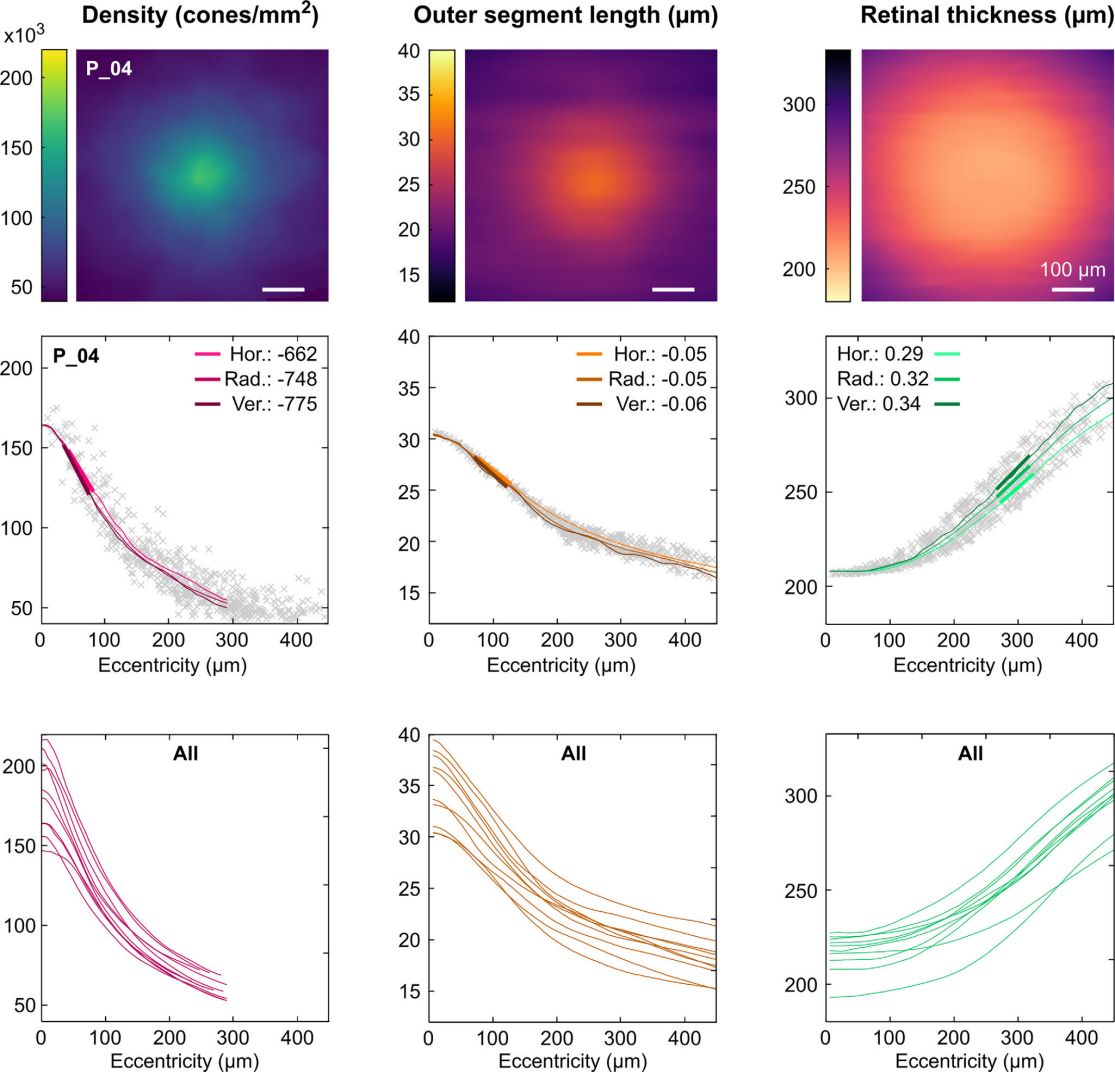
Cone density, OSL, and RT topography and slope analysis. (**Top row**) Example datasets in participant P_04, across the central ± 300 µm. (**Middle row**) One-dimensional view of cone density, OSL, and RT as a function of eccentricity. Numbers are the steepest slope for the horizontal, radial, and vertical average profiles. Grey crosses are data from individual cones. Although single cone densities could be analyzed at further eccentricities, average profiles of cone density were analyzed only until about 300 µm, owing to otherwise under-representation of densities along the cardinal meridians. (**Bottom row**) Individual radial average profiles shown for all 10 participants.

In addition to the comparison of singular maximum/minimum values, the full two-dimensional topography of cone density, OSL, and RT within the foveola was analyzed (Fig. 4). To that end, the horizontal, radial, and vertical average profiles of cone density, OSL, and RT were calculated from the two-dimensional maps. Cone density showed the steepest change over eccentricity with a maximal slope at approximately 50 µm eccentricity. The OSL slope was not as steep as cone density and had its maximum at approximately 100 µm eccentricity. Both density and OSL profiles had in common that the slope changed significantly at approximately 200 µm. Although density and OSL decrease steeply within the first 200 µm eccentricity, the RT is plateau shaped within the first 150 µm, reaching its maximum increase at approximately 300 µm eccentricity. Despite the large range in absolute numbers, the relative cone density, OSL, and RT profiles across eccentricities were quite similar across all 10 participants.

**Figure 5.**
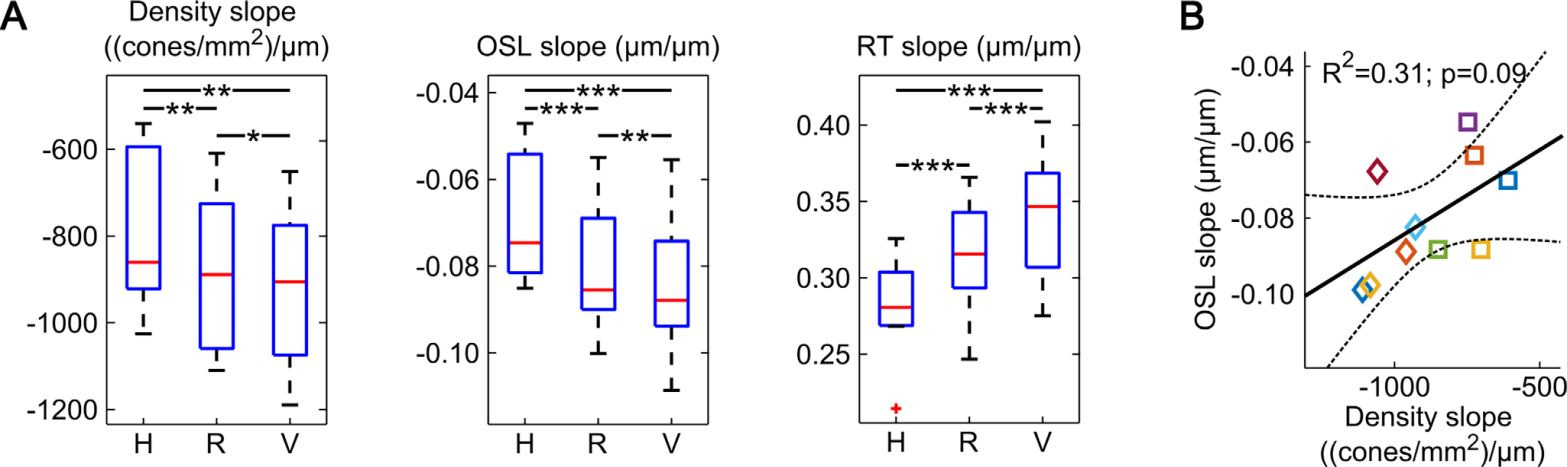
Horizontal–vertical anisotropy of foveal anatomy. (A) Comparison of the (H) horizontal, (R) radial, and (V) vertical topography slope averages reveals a significant anisotropy for all three tested anatomical parameters (paired *t* test; *P < 0.05; **P < 0.01; ***P < 0.001). (**B**) The steepest slope of the radial average OSL and radial average cone density showed the highest correlation, but no statistical significance. Participant markers are the same as in Figure 2. Dashed lines indicate confidence intervals.

A statistical comparison between the horizontal, radial, and vertical average profiles revealed a significant difference for the cardinal meridians. In 29 of 30 cases, the vertical profiles had the steepest slope, whereas the horizontal profiles were the shallowest (Fig. 5A). Furthermore, none of the slopes were significantly correlated. The strongest correlation was observed between cone density and OSL slopes (R^2^ = 0.31; *P* = 0.09) (Fig. 5B).

**Figure 6.**
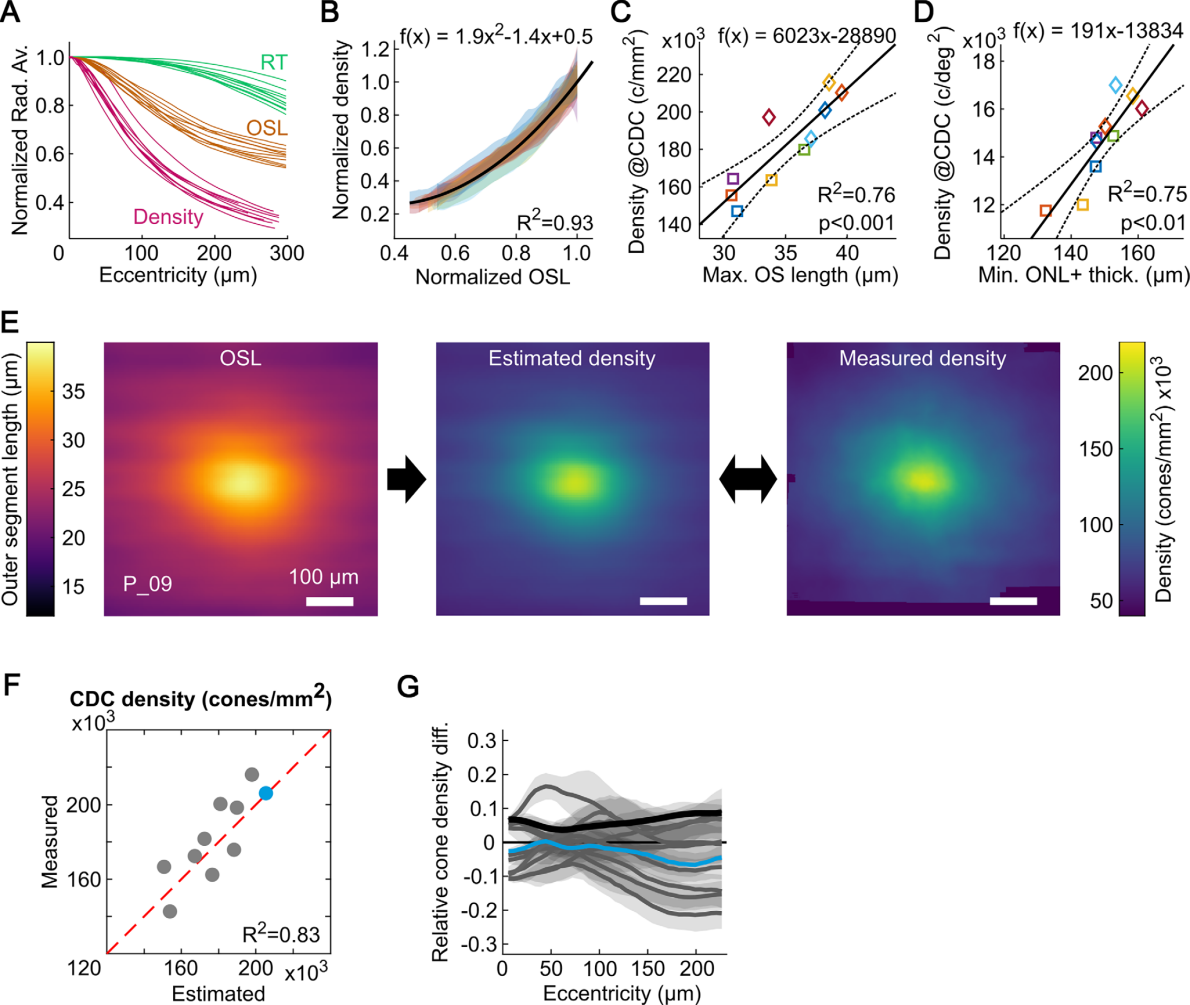
Inferring cone density from OCT-derived metrics. (**A**) When normalized to their maximum values, the foveal metrics used in this study display distinct features in their profiles, similar across participants. (**B**) The correlation between normalized cone density and normalized OSL was fitted by a quadratic function. (**C**) Linear regression function to estimate central cone density in linear units (cones/mm²) from maximum OSL. (**D**) Linear regression function to estimate central cone density in angular units (cones/deg²) from minimum ONL+ thickness. Participant markers in (**C**) and (**D**) are the same as in Fig. 2E. Exemplary application of an OSL-based foveolar cone density estimation and the AOSLO image-based cone density map in the fellow eye of P_09. (**F**) Comparison of measured and estimated central cone density yields an average difference of 11,580 cones/mm², or 6%, across all fellow eyes. (**G**) Quantitative analysis of the estimation quality given by the relative cone density difference comparing the radial average cone density profiles between measured and estimated data. The colored curve shows the relative difference for the example shown in (**E**), grey lines are all eyes. Grey shaded areas indicate the standard deviation for the relative difference at the given eccentricity. The average absolute difference was <10% across all participants and eccentricities (*black line*).

When normalized to their individual maximum value, cone density, OSL, and RT display distinct profiles, which are similar across participants (Fig. 6A). The relationship between normalized cone density and normalized OSL within the central ±300 µm was described best by a square function. The optimal fit was given by the following equation: *y* = 1.9*x*2 – 1.4*x* + 0.5, with *x* representing the normalized OSL at any foveolar location and y the according normalized cone density (Fig. 6B).

To estimate absolute cone density values from OCT data, a linear regression was applied describing the relationship between central cone density in linear units (cones/mm²) and maximum OSL. The resulting equation was, *y* = 6023*x* – 28,890 (Fig. 6C). For angular units of cone density (cones/deg²), the linear regression for the relationship between cone density and minimum ONL+ thickness yielded the best result (see Fig. 2), given by *y* = 191*x* – 13,834 (Fig. 6D). Combining the observed relationship between normalized cone density and normalized OSL with the linear regression between central cone density and maximum OSL, an estimation of foveolar cone density from foveal OCT data was possible following these three steps:

1) Normalization of the OSL map.
2) Conversion of the normalized OSL map into a normalized density map using *y* = 1.9*x*2 – 1.4*x* + 0.5.
3) Generation of the cone density in absolute units by multiplication of the normalized density map with the estimated CDC cone density from maximum OSL using y = 6023*x* – 28,890.

An exemplary application of this process is shown in Figure 6E, based on the fellow eye's data from P_09 (complete data set provided in Supplementary Fig. S2). Specifically, CD_CDC_ estimated from OSL was compared against measured density and yielded an average absolute difference of 11,539 cones/mm², or 6% (Fig. 6F). Additionally, estimated and measured fellow eye's cone density was compared across eccentricity in the two-dimensional maps. Across all participants, the average absolute difference was less than 8% within the inner 100 µm eccentricity and increased to approximately 10% at 200 µm eccentricity (Fig. 6G). In both cases, this is most likely an underestimation of the real estimation error, because the relative difference between measured and calculated cone density based on the found regression is approximately 5%, and because the fellow eyes ' OSL and density data are closely correlated (see Fig. 3).

## Discussion

In the present study, a significant correlation between foveolar cone density and OSL, as well as RT, was demonstrated from in vivo human retinal imaging. This enables prediction of the central cone mosaic in healthy eyes based on lower resolution clinical-grade OCT data. The best fit to estimate the CD_CDC_ in linear units from OCT-derived measurements of OSL was:

$$\begin{array}{c} {\mathrm{CD}}_{\mathrm{CDC}}\left({\mathrm{cones}/\mathrm{mm}}^{2}\right)=6023\cdot \mathrm{OSL}\left(m\right)-28,890. \end{array}$$

We also presented a more elaborate method to estimate full two-dimensional cone density maps from OCT data (see Fig. 6). However, owing to the limited sample size and high correlation between the analyzed fellow eyes, future studies need to assess the limitations and usability of the proposed estimation method. To note, based on the fellow eye's data set, the estimation of cone density in linear units was best when using the OSL (<7% difference), and slightly worse when using the ONL+ (<13% difference). To estimate cone density in angular units, the ONL+ produced the lowest difference between the estimation and data (<7% difference):

$$\begin{array}{c} {\mathrm{CD}}_{\mathrm{CDC}}\left({\mathrm{cones}/\deg}^{2}\right)=191\cdot \mathrm{ONL}+\left(m\right)-13,834. \end{array}$$

Based on observations from ex vivo data that the cone OS volume is relatively constant in a given retina,^16^ a direct relationship between cone OSL and cone density emerges. This finding is further backed by reports of a significant relationship between OSL and IS diameter observed ex vivo for a small number of retinae at different developmental stages.^28^^,^^29^ In recent studies using in vivo imaging, cone density could be shown to correlate to OCT data only indirectly, or in cases where the retinal tissue is massively altered. One study used the integral of the interdigitation zone and found a significant correlation with cone density, but, limited by the lateral resolution of the used AO device, only at the parafovea for eccentricities of more than 2°.^30^ A second study correlated the average ISOS layer thickness of approximately 250 OCT scans, corrected for age and sex, with the average histological cone density data.^31^ Correction for age and sex is important when matching against normative data, because it was found that cone density,^28^^,^^32^ as well as OSL,^33^^,^^34^ decrease with age, and RT depends on sex, with males tending to have a thicker retina.^35^^,^^36^ This approach was, however, unable to consider individual differences in cone density, which are shown to be significant (see next paragraph). In albinism, where the degree of fovealization varies, a strong positive correlation was observed between in vivo peak cone density and the thickness of the ONL.^37^ Other earlier in vivo studies failed to confirm a relationship of cone density and OSL in healthy participants.^19^^,^^20^ We explore here whether this may be due to the reliability with which such microscopic measurements can be performed in the living eye.

Our measurements of absolute cone density, OSL, and RT compare well with previous findings. It is worth noting that the CD_CDC_ (147,038 to 215,681 cones/mm²), was comparable with peak cone densities reported by other studies using adaptive optics ophthalmoscopy (e.g., 136,132 to 247,061 cones/mm² and approximately 130,000 to 200,000 cones/mm²),^4^^,^^38^ but significantly less ranged than measurements from explant retinas (100,000 up to 300,000 cones/mm²).^1^^,^^39^ An explanation could be tissue disruption and shrinkage during the histological preparation, leading to both under- and overestimation of cell density at the extremes.^8^^,^^40^ The measurement error for cone density maps generated from in vivo imaging is estimated to be approximately 11.75%.^41^ We find that cone density profiles were steeper for the vertical than the horizontal meridian, in good accordance with previous studies.^4^^,^^30^^,^^42^ Steeper density profiles within the first 150 µm with a distinct slope decrease at higher eccentricities is an observation visible in histological data as well,^1^ which adds general confidence in in vivo foveal topography data.

We observed maximum OSLs between 31 and 40 µm, close to histological data (30–50 µm),^16^^,^^43^^,^^44^ but shorter than earlier reports using spectral domain OCT images (41–53 µm).^19^^,^^33^^,^^45^ This difference is likely due to an earlier assumption about the cellular origin of the outer OCT bands, leading to an OSL overestimation in former studies.^27^ Additionally, the higher resolution of the OCT instrument used in our study might have played a role as well, because the increased signal quality helps to distinguish between the two OSL-defining bands. Bending or coiling of the OS as it is sometimes observed in histological preparations cannot be ruled out and corrected for, which would result in an underestimation of the actual OSL. We observed a minimum RT in the range of 193 to 226 µm, which is in good accordance with previous studies reporting the central point thickness from spectral domain OCT images of approximately 270 µm,^46^ 231 µm,^47^ and 214 µm.^48^ In histological preparations, the average central RT was found to be about 205 µm,^49^ close to our average of 218 µm.

The repeatability of OSL and RT extraction from OCT measurements was tested on one participant (P_06) of this study by recording additional OCT images on 4 days consecutively (Supplementary Fig. S3). The OCT raw image data (.vol files) were processed through the same analysis pipeline and the variability of maximum OSL, as well as minimum RT, and ONL+, measurement was computed. The standard deviation of these five measurements was ±0.6 µm for OSL and ONL+ and ±1.4 µm for RT.

Despite similarities in group averages, it remains questionable why earlier studies failed to observe a clear relationship between cone density and OSL. Limited axial resolution of the OCT devices used may be one factor: currently available clinical-grade spectral domain OCT devices have an axial resolution of approximately 5 µm per pixel,^50^ such that only a few image pixels correspond with the full length of the OSs. Similar to what we observed in our study, OSL standard deviation was reported to be 4 µm, close to the resolution limit of the former devices. Likely limited in such way, one study reported a significant correlation between cone spacing and OSL at 1°, 2°, and 3° eccentricity but not for cone spacing z-scores or the central foveola.^51^ Another study tried to circumvent the axial resolution limitation by evaluating the thickness of the OS plus the retinal pigment epithelium (OS+) and found a significant correlation between OS+ thickness and cone spacing z-scores at the foveal center.^52^ The prototype high-resolution spectral domain OCT system used here had an axial resolution of approximately 2 µm, less than one-half of standard systems, which may be required to capture the minuscule differences in OSL across individual human foveolas and retinal eccentricity.

Based on the relationships visible in our data and the assumption of a fixed OS volume, it is possible to calculate such volume. Assuming the foveolar cone packing as an ideal hexagonal mosaic, the area of each cone's IS visible in the AOSLO en-face images, A_IS_, is given by the equation,

(1) $$\begin{array}{c} A_{IS}=2*\sqrt{3}*r_{IS}^{2}, \end{array}$$

with *r_IS_* being the cone IS radius. The relationship between cone density and *r_IS_* is thus given by the reciprocal:

(2) $$\begin{array}{c} \mathrm{Density}=\frac{1}{2*\sqrt{3}*r_{IS}^{2}}. \end{array}$$

If the OS is considered a cylinder, its volume is given by,

(3) $$\begin{array}{c} V_{OS}=\pi *r_{OS}^{2}*OSL. \end{array}$$

The transition from the inner to OS can be described by the ratio *R_OSIS_*:

(4) $$\begin{array}{c} R_{OSIS}=r_{OS}/r_{IS}. \end{array}$$

Including this transition between IS and OS radii, the OS volume can be calculated from the IS radius:

(5) $$\begin{array}{c} V_{OS}=\;\pi *{\left(R_{OSIS}*r_{IS}\right)}^{2}*OSL. \end{array}$$

Because cone density is defined by the IS’ radius (Equation (2)), the OS volume can be written as:

(6) $$\begin{array}{c} V_{OS}=k*\frac{OSL}{Density},\;with\;k=\frac{\pi *{\left(R_{OSIS}\right)}^{2}}{\sqrt{3}*2}. \end{array}$$

If *V_OS_* and *R_OSIS_* are constant, the relation between OSL and cone density is linear, as it was observed here for the maximum OSL in the central foveola (Fig. 2A). The slope of the linear regression fitted to the group data was m = 126 µm^3^. Using a fixed value of 0.66 for *R_OSIS_*,^19^ we arrive at an average OS volume of 50 µm^3^ across all our participants. If we assume constant volume within a single retina, calculation of the OS volume for each participant yielded a range of OS volumes between 68 and 83 µm^3^. Literature reports similar OS volumes varying between 32 to 47 µm^3^ and 141 µm^3^ for human cones^28^^,^^44^ and 86 µm^3^ for macaque cones.^16^ A deviation from a linear relation between cone OSL and cone density, as it was observed here within an individual eye across eccentricities, could be the result of a variable *R_OSIS_*, where the transition of IS to OS radii is a function of the cone's packing density. In this case, a quadratic relationship applies (compare Fig. 6B). A varying OS volume would result in a nonlinear relation between OSL and cone density.

We also observed a significant correlation between RT (or ONL+) and cone density in the foveal center. Although indications of such a relationship exists in the literature, it has not been demonstrated for the foveola before. A similar relationship was found for an individual retina across eccentricity, but not across individual eyes.^53^ RT seems to be correlated with the diameter of the foveal pit^54^ as well as the effective diameter of the foveolar avascular zone.^47^ However, in case of hypoplasia (e.g., in persons with albinism) foveal RT is much higher owing to the complete displacement of inner retinal layer, such that in an abnormal retina RT and foveolar avascular zone are no longer correlated.^55^ ONL thickness and cone density expressed in angular units (cones/deg²) at eccentricities larger than 1° was shown to correlate in healthy eyes as well as retinitis pigmentosa patients.^56^ Other studies examining diseased eyes found that OSL reduction for patients with inherited retinal diseases is correlated with a loss of cones.^57^ Patients recovering from chorioretinopathy showed an increase of macular cone density, correlated with an increase in the outer retinal layer thickness.^58^

We found a quadratic relationship between normalized cone densities and normalized OSL within the full radius of the foveolar center, very similar across our participants, despite larger differences in absolute numbers (see Fig. 6B). We also found a linear relationship between the total numbers of cones within the central 1° diameter and the central cone density, CD_CDC_ (data not shown). Both observations suggest that the fovea is formed under the influence of similar factors in each eye, but with a different total number of cones at the beginning of foveal maturation. This may suggest the presence of a common blueprint orchestrating the complex series of events that start during pregnancy and end in childhood forming the fovea.^28^^,^^59^^,^^60^ Zhang et al.^38^ found that the total number of cones within 1 mm eccentricity was constant, which allows formulation of the hypothesis that the central fovea forms with different cone migration rates in each individual. The existence of a singular foveal growth factor, is—to our best knowledge—unknown.

The centripetal cone migration during foveal maturation is completed at an age of approximately 5 years,^61^ similar to the onset age of myopia.^62^ What remains unclear is how the shape of the eyeball changes and how retinal morphology adapts to the additional eye growth.^63^^,^^64^ Here, we observed a tendency toward decreased cone density in linear units (cones/mm²) with increasing axial length (see Table), whereas the angular cone density (cones/deg²) does vary less with axial length. Such observation supports the hypothesis of a global expansion rather than equatorial stretching or an overdevelopment of cones to fill in the increasing space. Following the global expansion model, the tightly squeezed cone photoreceptors expand into the additional space during myopic growth. In turn, the OSs would become wider and shorten, based on the volume constancy, resulting in a close relation of linear cone density and OSL, but not for angular cone density. We here observed a highly significant correlation between OSL and linear cone density (R² = 0.7) and a less strong correlation between OSL and angular cone density (R² = 0.4). This observation supports the idea of a mixture between the global expansion and equatorial stretching model, as it was reported before when comparing linear and angular cone densities.^63^

Direct quantification of the foveal cone mosaic and its pathological structural changes is a powerful tool for ophthalmologists to detect early onsets of retinal diseases, ideally before the visual system is affected on a perceptual level: for example, in patients with retinitis pigmentosa, structural degradation such as decreasing cone density (or increased cone spacing) was found to precede functional degradation.^12^ Visual acuity, a critical biomarker in clinical studies, may be less sensitive, as it was found to be reduced only after cone density was reduced by 40% or more.^57^ Fine structural analysis thus opens a window of opportunity for potential therapeutical intervention before visual function is lost. Unfortunately, retinal imaging systems capable of resolving foveal cones are expensive, complex to maintain and operate, and require optimal imaging conditions. The latter may be difficult to achieve routinely, especially in the case of the aging eye and in the presence of retinal disease which are often associated with insufficient ocular wavefront correction and/or abnormal eye motion (e.g., nystagmus^65^). Therefore, estimation of foveal cone density from OCT images could help to circumvent such limitations by lifting the need to resolve the foveal cone mosaic directly. The relationship between OCT-derived measurements of OSL and RT, and cellular-resolved measurements of cone density shown here may help to guide future clinical studies focusing on structural changes of the foveal center.

## Supplementary Material

Supplement 1
