# Supplementary material for: Cone Density Is Correlated to Outer Segment Length and Retinal Thickness in the Human Foveola
Source: Invest Ophthalmol Vis Sci. 2023 Dec 8;64(15):11. doi: 10.1167/iovs.64.15.11 (PMC10709802; doi:10.1167/iovs.64.15.11)
Supplement: Supplement 1 [file iovs-64-15-11_s001.pdf]

Manuscript title: Cone density is correlated to outer segment length and retinal thickness in the human foveola

Supplemental

Authors: Niklas Domdei[1], Julius Ameln[2], Aleksandr Gutnikov[2], Jenny L. Witten[2], Frank G. Holz[2], Siegfried Wahl[1,3], Wolf M. Harmening[2]

Affiliations:
1 Carl Zeiss Vision International GmbH, Aalen, Germany
2 Department of Ophthalmology, University of Bonn, Bonn, Germany
3 Institute for Ophthalmic Research, Eberhard Karls University Tübingen, Tübingen, Germany

Corresponding author:
Wolf Harmening, wolf.harmening@ukbonn.de

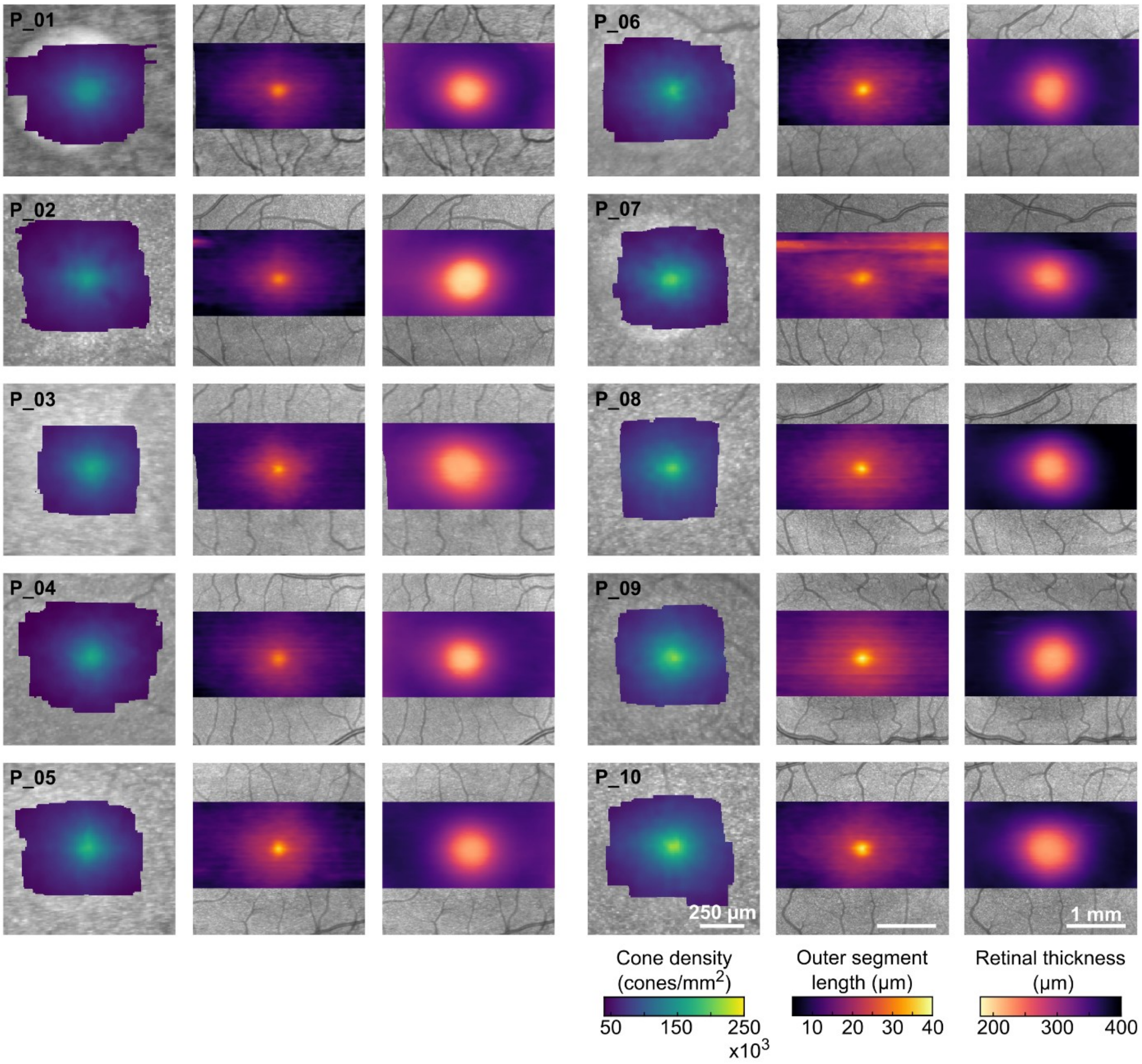

Figure S1: Two-dimensional retinal maps of cone density (left), outer segment length (middle) and retinal thickness (right columns), in the dominant eyes of all participants (P_01-10). Participants were named in an ascending order of their cone density at the CDC (P_01: 147,038 cones/mm^2; P_10: 215,681 cones/mm^2). The high OSL readings for P_07 outside the foveola (and hence outside the analyzed area) are the result of segmentation artefacts.

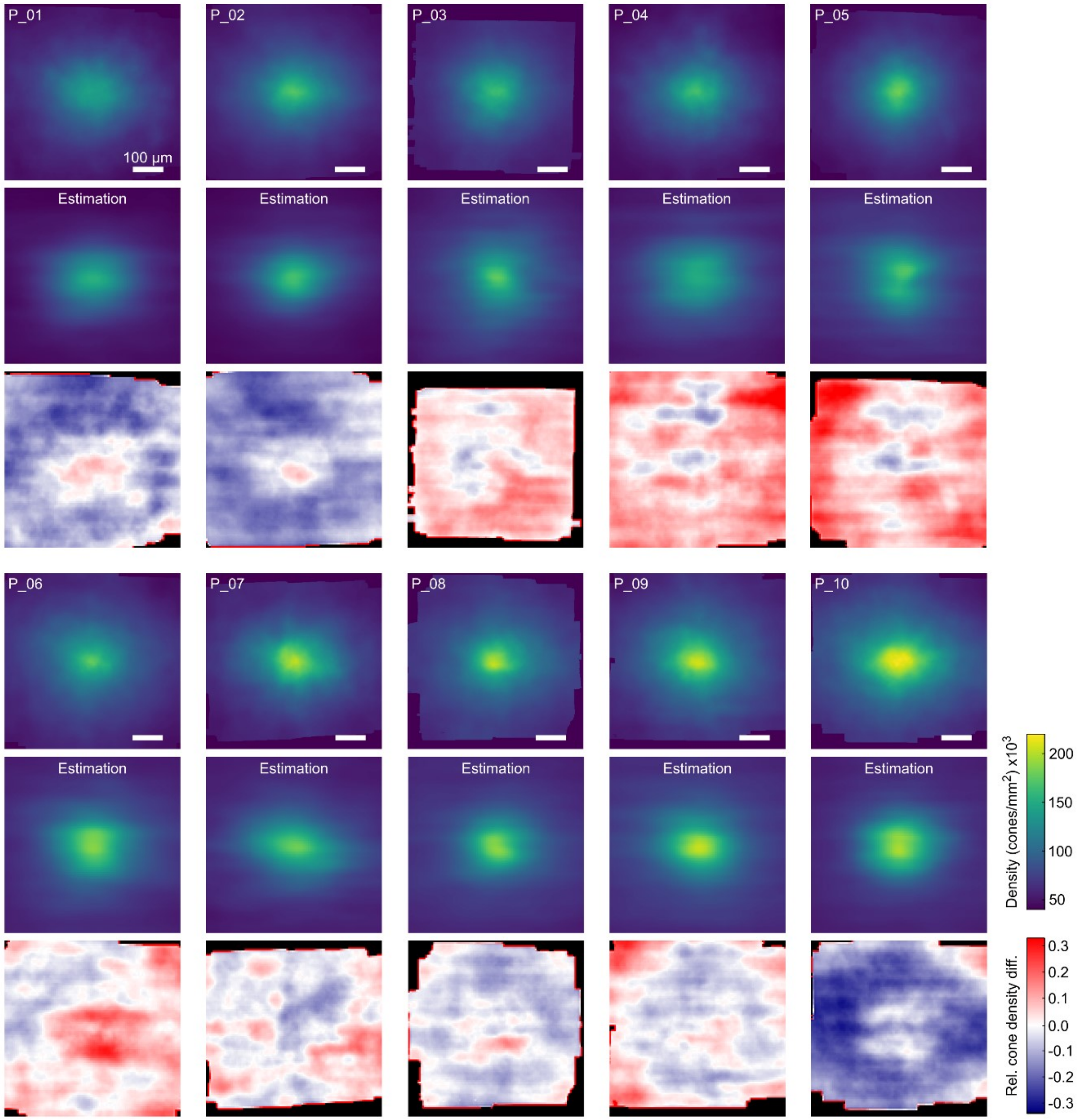

Figure S2: Two-dimensional retinal maps of cone density, cone density estimation, and difference between counted and estimated, for the non-dominant eyes of all participants.

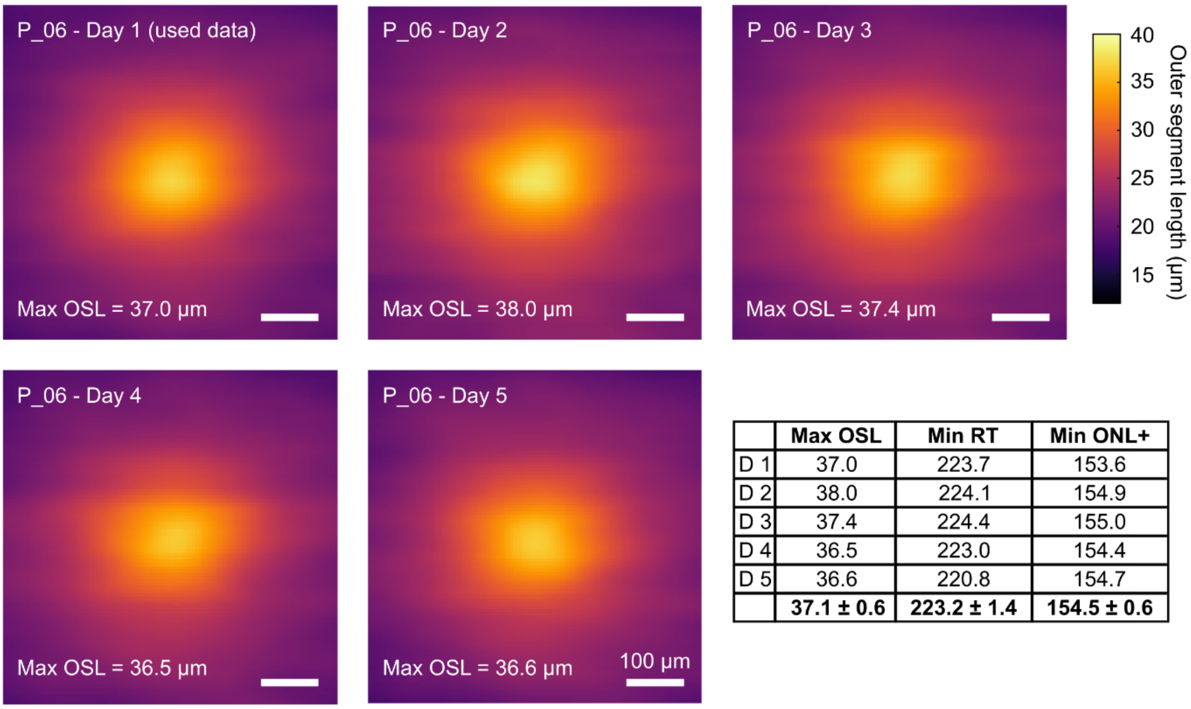

	Max OSL	Min RT	Min ONL+
D 1	37.0	223.7	153.6
D 2	38.0	224.1	154.9
D 3	37.4	224.4	155.0
D 4	36.5	223.0	154.4
D 5	36.6	220.8	154.7
	**37.1 ± 0.6**	**223.2 ± 1.4**	**154.5 ± 0.6**

Figure S3: Variability analysis for repeated OCT derived OSL maps of the same participant. Additional OCT images were recorded across 4 more days. The table shows the summary for maximum OSL as well as minimum RT and Min ONL+ in µm for these 5 days with the resulting average ± STD in the last row.
